# Attachment Patterns in Subjects Diagnosed With a Substance Use Disorder: A Comparison of Patients in Outpatient Treatment and Patients in Therapeutic Communities

**DOI:** 10.3389/fpsyt.2019.00807

**Published:** 2019-11-06

**Authors:** Laura Vismara, Fabio Presaghi, Maria Bocchia, Rosolino Vico Ricci, Massimo Ammaniti

**Affiliations:** ^1^Department of Educational Sciences, Psychology, Philosophy, University of Cagliari, Cagliari, Italy; ^2^Department of Psychology of Development and Socialization Processes, Sapienza University of Rome, Rome, Italy; ^3^Department of Mental Health (DSM), SERT, Local Health Service of Sarzana DSS 17, Sarzana, Italy

**Keywords:** substance use disorder, attachment patterns, care system, diagnosis, intervention

## Abstract

The purpose of the present study is to analyze the quality of attachment in substance abuse patients in outpatient treatment vs. patients in therapeutic communities in order to identify the role of attachment insecurity in choosing a care system. The sample consisted of 127 subjects (107 males and 20 females); 97 were outpatients (83 males) and 30 therapeutic community patients (24 males). Attachment with respect to current, significant relationships was assessed using the Relationship Questionnaire. In the outpatient subgroup, the prevailing attachment style was preoccupied; for the therapeutic community patients, the prevailing attachment style was dismissive. The dimensions of care (how the caregiver is perceived as loving and caring) and overprotection (how the caregiver is perceived as intrusive and interfering)—evaluated by means of the Parent Bonding Instrument—were higher in the outpatient subgroup. Scores were higher with respect to maternal subscales regardless of treatment modality. No differences emerged with respect to self-perceived symptoms (SCL-90-R) between the subgroups; however, fearful-avoidant and dismissive-avoidant individuals reported higher self-perceived symptom regardless of treatment modality. Understanding the distribution of different attachment patterns with respect to the treatment modality may improve efficacious interventions, attuning them to the individual and his or her developmental environment.

## Introduction

Substance abuse is a relevant phenomenon at a clinical and social level in Western countries: about 50.0% of youths use illicit substances by age 16 (1–3). Among the complex interaction of variables that may contribute to such a phenomenon (4) the contribution of family experiences will be the focus of the current study.

In the context of substance abuse and dependence, family relations are found to lack support and be disorganized, multi-problematic, unpredictable, and inconsistent (5–10). It stands to reason that such experiences impact the attachment system and, consequently, the development of emotional regulation and self-representation (11–14). Indeed, a vulnerable self-regulation system is one of the most significant risk factors for substance abuse and dependence (15–17).

Despite the importance of attachment theory to the mechanisms linked to the onset of substance dependence and abuse, research on the subject is still limited. Existing empirical data have shown a link between first attachment relations and subsequent development of a dependence disorder (12, 18–22). Data have also confirmed the role of attachment in the context of substance use disorders (SUDs), not only at a behavioral and representational level but also at a neuronal level, demonstrating decreased white matter connectivity in poly-drug users (23, 24). However, data do not explain the direction of the influence of attachment and substance abuse.

Differences concerning prevailing attachment patterns may be due to the heterogeneity of the adopted methods. In fact, much of the current data are derived from studies conducted on clinical, but not select, groups. These examined subjects may present a primary diagnosis other than substance abuse, and many show a high incidence of comorbidity. In addition, attachment patterns may vary in relation to the kind of used substance (25).

Another discriminating aspect concerns the chosen instrument. Some studies applied the Adult Attachment Interview (AAI) (26). Others used self-reports that defined different models of attachment based on different, specific assumptions (27–29).

Within AAI’s studies, the majority of the subjects showed either dismissive or enmeshed-preoccupied insecure attachments (19, 21, 30). Fonagy et al. (19) found the unresolved-disoriented attachment pattern to be the most frequent, showing the inability to process traumatic experiences as a crucial variable for the onset of such disorders.

Studies that applied Hazan and Shaver’s self-report questionnaire (31) indicated avoidant attachment as the most common style among substance users (32, 33). Using Bartholomew’s four categories of attachment (34, 35), the prevalent attachment strategy was either dismissive-avoidant or fearful-avoidant (29, 36, 37). Schindler et al. (38) carried out a cluster analysis to show the family attachment patterns of its members. The majority of members showed a “triangulated” pattern: preoccupied mothers, dismissive-avoidant fathers, and fearful adolescents.

Moreover, differences may depend on comorbidity. Several studies have shown a high association between substance abuse and personality disorders (39). Also, more negative consequences have emerged in patients with a diagnosis of SUD and a comorbid major depressive or post-traumatic stress disorder (40).

Poly-substance abuse may also explain these empirical inconsistencies. Several studies have shown a high frequency of psychopathology among poly-abusers (39). However, the type of used substance does not seem to be linked to the degree of impairment in the attachment system or the personality disorder specifically (41).

In conclusion, substance abuse is associated with insecure attachment; however, it is not associated with a specific quality of insecure attachment. The current study contributes to the study of attachment in poly-substance abusers with respect to different treatment modalities: subjects in outpatient care vs. subjects in therapeutic communities.

Outpatient treatment deals with prevention, care, and rehabilitation. The main aim is to prevent the diffusion of legal and illegal substance abuse and to intervene in favor of the health of individuals and their families. *Therapeutic communities*, in comparison, carry out personalized therapeutic interventions in a residential context.

Several studies have looked at the efficacy of interventions with substance-dependent individuals (42, 43). Meta-analytic reviews have shown that there is no substantial difference in treatment typology: hospital, therapeutic community, intensive, or ordinary outpatient treatment (44, 45). However, in the case of more serious diagnoses, hospitalization seems most effective, while outpatient treatment seems more appropriate for patients with stable psychosocial conditions and minor impairments (46, 47).

Research (although not specifically focused on substance abuse) has shown that patients in therapeutic communities often have more vulnerable backgrounds. They come from mono-parental families, have experienced abuse, and exhibit more criminal behaviors, more depressive symptoms, alcoholism, more aggressive attitudes, and cognitions. Outpatients, in comparison, have more problems concerning medical and psychiatric comorbidity (48–50).

Understanding individuals’ attachment quality may help to establish a good treatment compliance that considers the specific individual and his or her family’s characteristics and problems (37, 51–55). Studies on the association between attachment patterns and treatment compliance are insufficient; yet, dismissive and avoidant styles seem to be the strongest connection to poor intervention outcome and adherence (56). One study targeting attachment in inpatients with a substance use diagnosis showed that anxious-preoccupied attachment was linked to treatment retention (57). However, other variables should be considered to explain the relationship between attachment and SUD treatment, such as comorbid personality disorder, cognitive deficits, and age (58, 59).

### Hypotheses

The purpose of the present study was to analyze the quality of attachment in subjects diagnosed with a SUD attending outpatient care compared to those attending therapeutic communities in order to identify the role of attachment in choosing treatment modality.

In particular, we expect:

A higher frequency of insecure attachment patterns compared to secure ones among subjects diagnosed with a SUD.

#### A Different Distribution of Attachment With Respect to Treatment Modality; Specifically:

– a higher frequency of dismissive-avoidant subjects—characterized by a predisposition to withdraw from family relationships—among subjects in therapeutic community treatment;
– a higher frequency of preoccupied subjects—characterized by a tendency to be over-involved in their family relations, from whom they are not able to become autonomous—among subjects in outpatient care.

#### A Different Family History With Respect to Treatment Modality; Specifically:

– a higher frequency of bonds—characterized by low care and low overprotection—among subjects in therapeutic community care, considering the absence or weakness of their relationship with family figures;
– a higher frequency of bonds—characterized by high or low care and high overprotection—among subjects in outpatient care, considering the controlling relationship with their family figures.
– a higher frequency of self-reported symptoms among subjects with an insecure attachment pattern, regardless of treatment modality.

## Materials and Methods

### Participants

A total sample of 127 subjects with a diagnosis of SUD (107 males and 20 females) were recruited in Liguria (northern region of Italy). There were n = 97 (83 males) outpatient participants and n = 30 (24 males) participants treated in *therapeutic communities* (**Table 1**). No relationship was found between gender and type of care system [χ ^2^(1) = .535 p > .05]. The average age of the participants was about 30 years (SD = 6.4, age range: 18 to 52). No significant age difference emerged between males (M = 30.28; SD = 6.29) and females (M = 29.30; SD = 6.80). Additionally, no differences emerged between subjects with a diagnosis of SUD attending outpatient care (M = 30.09; SD = 6.44) and those attending *therapeutic communities* (M = 30.23; SD = 6.17).

**Table 1:** Gender distribution with respect to care system.

	M		F		Total	
	Fr	%	Fr	%	Fr	%
Outpatient care	83	65.4	14	11.0	97	76.4
Therapeutic community care	24	18.9	6	4.7	30	23.6
Total	107	84.3	20	15.7	127	100.0

Considering the type of abused substance, about 75.8% of the sample (n = 75) reported heroin as their primary abused substance. The other abused substances included cannabinoids, cocaine, and ecstasy. No significant relationship emerged between the type of abused substance (heroin vs. other abused substances) and the type of chosen care system [χ ^2^ (1) = 0.01, p > .05, n = 99]. For 28 participants, it was not possible to determine the primary abused substance.

### Measures

The following battery of questionnaires was administered: the Symptom Checklist-90-Revised (SCL-90-R), the Relationship Questionnaire (RQ), and the Parental Bonding Instrument (PBI).

The SCL-90-R (1977/83) is a 90-item self-report that evaluates several psychological problems and symptoms. Items are scored on a scale from 0 (none) to 4 (very much), with respect to nine symptom scales: SOM (somatization), O-C (obsessive-compulsive), I-S (interpersonal sensitivity), DEP (depression), ANX (anxiety), HOS (hostility), PHOB (phobic anxiety), PAR (paranoid ideation), and PSY (psychoticism). Global indexes refer to the Global Severity Index (GSI), which measures overall psychological distress; the Positive Symptom Distress Index (PSDI), which measures the intensity of symptoms; and the Positive Symptom Total (PST), which reports a number of self-reported symptoms. The SCL-90-R has shown good convergent validity with the MMPI (60) and with the GHQ-28 (61). Test-rest reliability indexes are also satisfying, ranging from .68 (somatization) to .83 (paranoid ideation) with an interval of 2 weeks (62).

The RQ (63) consists of a single item that describes each of the four-category representations of attachment in close relationships (i.e., secure, preoccupied, fearful-avoidant, and dismissive-avoidant) in four short paragraphs. Respondents rate their degree of correspondence with each description (**Table 2**) on a 7-point scale.

**Table 2:** Bartholomew and Horowitz’s model of attachment relationships (63).

		Self (*Dependence*)	
		*Positive*	*Negative*
Other *(avoidance)*	*Positive*	SECURE At ease with intimacy and autonomy	PREOCCUPIED Preoccupied by relationships
	*Negative*	DISMISSING/AVOIDANT Refusal of intimacy and dependence	FEARFUL/AVOIDANT Fear of intimacy and social avoidance

The RQ allows for both a categorical and a dimensional evaluation of a subject. With respect to the latter, individuals may be described along two dimensions: a) self model/anxiety and b) other model/avoidance. Inter-rater reliability ranged from .87 and .95 (64), while convergent validity was satisfactory, considering the AAI three-category system (65). The test-rest reliability was also discrete (about 70.0% of congruent classifications) after a 4-year interval (66).

The PBI (67; 68) is a 25-item self-report that evaluates maternal and paternal care and over-protection during the first 16 years of a child’s life. The 12 “care” and 13 “over-protection” items are rated on a 4-point Likert scale from 0 (not at all) to 3 (completely). The combined (high vs. low) score allows a researcher to attribute one of the four attachment categories (**Table 3**). Cut-off scores for the care dimension are 27 and 24 for the mother and father versions, respectively; cut-off scores for over-protection are 13.5 and 12.5 for the mother and father versions, respectively. The PBI showed a good construct and convergent validity (67) as well as good test–retest reliability, ranging from .79 to .96 (69).

**Table 3:** Parenting styles according to Parker et al.’s model (66).

	High overprotection	Low overprotection
**High care**	Affectionate constraint	Optimal bond
**Low care**	Affectionless control	Weak bond

### Procedure

Participants were recruited within the public health service of La Spezia (Italy). Instruments were administered within the clients’ evaluation/intervention program, for which patients signed written consent.

Attachment measures were added to the standard evaluation process carried out by the Local Health Service; it involved clinical interviews, the Structured Clinical Interview for the DSM-IV (SCID-IV, 1994), and the MMPI-2 (70). Regular medical drug testing was also performed. Diagnoses, therefore, were provided to the research team by the Local Health Service.

The study was approved by the Ethical Committee of the University of Cagliari (prot. N° 2019-UNCACLE-0228682).

### Overview of Statistical Analysis

Chi-square statistics were used to investigate the relationship between the distribution of attachment categories and to inspect the direction of the relationship we considered standardized residuals. Analysis of variance and analysis of covariance were considered for investigating average differences among the PBI dimensions and psychopathological distress assessed with SCL-90-R with respect to the care system and attachment categories. For ANOVA we will consider estimates of partial eta squared as measure of effect sizes assuming values around .01 as “small” effect size, values around .09 as “medium” effect size, and values around .25 as “large” effect size. Finally, a logistic regression analysis was used to investigate whether attachment categories and dimensions reliably predict the care system choice.

## Results

### Distribution of Attachment Categories as Function of the RQ and PBI

**Table 4** shows the distribution of attachment categories in the sample of subjects diagnosed with a SUD based on the RQ classification system. As expected, there was no equal distribution between the four attachment categories [*χ* *^2^* (3) = 29.05, *p* < .01] and the most frequent category was preoccupied attachment (43.7%) while fearful-avoidant attachment was the least represented (10.3%). As no comparison group was available, we compared this distribution of attachment categories to a similar sample examined by Schindler et al. (54) although constituted by adolescents (**Table 4**).

**Table 4:** Distribution of the Relationship Questionnaire attachment categories (Secure Vs Preoccupied Vs Fearful/Avoidant Vs Dismissing/Avoidant) by nationality (Germans vs. Italians) and care system (outpatients vs. residentials).

	German Sample^a^		Italian Sample					
			*Total* *^b^*		*Outpatients*		*Residentials*	
	*Fr*	*%*	*Fr.*	*%*	*Fr.*	*%*	*Fr.*	*%*
Secure	5	6.0	31	24.6	25	26.0	6	20.0
Preoccupied	12	17.0	55	43.7	47	49.0	8	26.7
Fearful/avoidant	46	65.0	13	10.3	9	9.4	4	13.3
Dismissing/avoidant	8	11.0	27	21.4	15	15.6	12	40.0
Total	71	100	126	100	96	100	30	100

^a^Data retrieved from Schindler et al (54); ^b^ the care system was unknown for one participant

Secure, Secure attachment category; Preoccupied, Preoccupied attachment category; Fearful/Avoidant, Fearful/Avoidant attachment category; Dismissing/Avoidant, Dismissing/Avoidant attachment category; Outpatients, Patients attending public care service; Residentials, Patients attending the therapeutic community service.

The two distributions (Italian adults vs. German adolescents) diverged systematically [*χ* *^2^* (3)= 64.8, *p* < .01] with respect to the fearful-avoidant attachment category that was significantly less frequent in the Italian sample (10.3%) than in the German sample (65.0%). This difference remained even when we excluded participants who chose *therapeutic communities* from the Italian sample [*χ* *^2^* (3)= 58.7, *p* < .01; 9.0% of Italian fearful-avoidant], or when we excluded those who chose to attend outpatient services [*χ* *^2^* (^3^) = 24.3, *p* < .01; 13.0% of Italian with fearful-avoidant attachment]. Moreover, the two Italian distributions (outpatient-treated and *therapeutic community–treated*) were different from each other [*χ* *^2^* (3) = 9.6, *p* < .05]; dismissive-avoidant attachment was more frequently observed in the *therapeutic community*-treated group (40.0%, with respect to the 16.0% observed in the outpatient group).

Next, we considered the distribution of the attachment categories (**Table 5**) obtained from the PBI cut-off scores; first, separately for each version (mother vs. father) and then successively in combination. The distribution of attachment categories for the mother version of PBI was characterized by an over-representation of the category “affectionless control” [*χ* *^2^* (3) = 25.09, *p* < .05, approximately 44.4% of the total sample]. In comparison, the distribution of the father version was characterized by an under-representation of the category “affectionate constraint” [*χ* *^2^* (3) = 18.06, *p* < .05, respectively 11.1%]. The two distributions (the PBI category, mother version vs. the PBI category, father version) were moderately correlated [Pearson contingency correlation coefficient = .521, *χ* *^2^* (9) = 48.87, *p* <.01].

**Table 5:** Classification of subjects diagnosed with substance use disorder with respect to Parental Bonding Instrument’s (PBI) maternal and paternal cut off scores.

	Father								Tot
	Affectionate constraint		Optimal Bond		Optimal Bond		**Weak or Absent Bond**		
	Fr	%	Fr	%	Fr	%	Fr	%	
**Mother**									
**Affectionate constraint**	3	2.4	12	9.5	9	7.1	1	0.8	25
**Affectionless control**	8	6.3	24	19.0	2	1.6	22	17.5	56
**Optimal Bond**	2	1.6	3	2.4	12	9.5	4	3.2	21
**Weak or Absent Bond**	1	0.8	4	3.2	4	3.2	15	11.9	24
**Total**	14	11.1	43	34.1	27	21.4	42	33.3	126

Considering the distribution of PBI attachment categories with respect to the attended care system (**Table 6**), a significant relationship for the mother version of the PBI [*χ* *^2^* (3) = 16.28, *p* < .01] emerged in which subjects with a diagnosis of SUD with a “weak or absent bond” more frequently chose to attend a therapeutic community (10.2%, standardized residual = 3.9) rather than an outpatient clinic (8.7%, standardized residual = -3.9). Such a difference did not emerge in relation to the father version [*χ* *^2^* (3) = 5.38, *p* > .05].

**Table 6:** Contingency table of attachment patterns according to maternal and paternal Parental Bonding Instrument (PBI: Optimal Bond vs Weak or Absent Bond vs Affectionless control vs Affectionate constraint) and care system (outpatients. vs residentials).

	Mother				**Father**			
	Outpatients		Outpatients		Outpatients		Outpatients	
	Fr	%	Fr	%	Fr	%	Fr	%
*Optimal Bond*	16	12.6	5	4.0	23	18.2	4	3.2
*Weak or Absent Bond*	11	8.7	13	10.2	27	21.4	15	11.9
*Affectionless control*	47	37.0	9	7.1	34	27.0	9	7.1
*Affectionate constraint*	23	18.1	3	2.3	12	9.6	2	1.6
Total	97	76.4	30	23.6	96	76.2	30	23.8

Nevertheless, it is important to emphasize that even if our data show that people treated in therapeutic communities are dismissive-avoidant (*n* = 12) in the RQ, and show a weak or absent bond to the mother (*n* = 13) in the PBI, RQ, and PBI subjects belonging to such groups are not necessarily the same individuals. Specifically, only four residentials are simultaneously dismissive-avoidant in the RQ and show a weak or absent bond to the mother in the PBI.

### Average Differences Among PBI Dimensions With Respect to the Care System

The scores on the care and protection dimensions of the PBI (mother vs. father), reported by those who had chosen outpatient care rather than therapeutic community care, were successively compared in an ANCOVA (2 × 2) with the SCL-90-R anxiety and depression scores as covariates. The latter was performed in order to exclude the potential effect of affective symptoms contributing to the choice of the attended care system.

Results showed a significant main effect of the care system factor [*F* (1, 115) = 4.66, *p* <. 05, *η* ^2^ *_P_* = .04] where subjects outpatient SUD reported higher average levels of care (*M* = 19.98) with respect to those attending therapeutic communities (*M* = 16.40). A significant main effect of the PBI version factor was also found [*F* (1, 115) = 10.21, *p* <. 01, *η* ^2^ *_P_* = .08] where higher levels of care for the mother version (*M* = 20.42) compared to father version (*M* = 16.40) were reported. Finally, the interaction effect was not significant [*F* (1, 115) = 1.86, *p* = .17]; neither were the effects of covariate [respectively: anxiety: *F* (1, 115) = 0.34, *p* = .56; depression: *F* (1, 115) = 1.22, *p* = .27].

The same 2 × 2 ANCOVA design was repeated, considering overprotection as the dependent variable. For this analysis, the two main effects of the care system factor and the PBI parent version produced significant results [respectively: *F*(1, 115) = 9.12, *p* < .01, *η* ^2^ *_P_* = .07; and *F*(1, 115) = 4.55, *p* < .05, *η* ^2^ *_P_* = .04]. The outpatient SUD reported higher levels of overprotection (*M* = 18.26), in contrast to the average seen in the therapeutic community care group (*M* = 14.23). Furthermore, the average overprotection was higher for the mother version of the PBI (*M* = 17.40) than the father version (*M* = 15.10). The interaction effect was barely significant [*F*(1, 115) = 3.55, *p* = .06, *η* ^2^ *_P_* = .03], and, in this case, the effects of covariates were largely insignificant [respectively, anxiety: *F* (1, 115) = 0.06, *p* = .81; depression: *F* (1, 115) = 0.44, *p* = .51].

**Figure 1:**
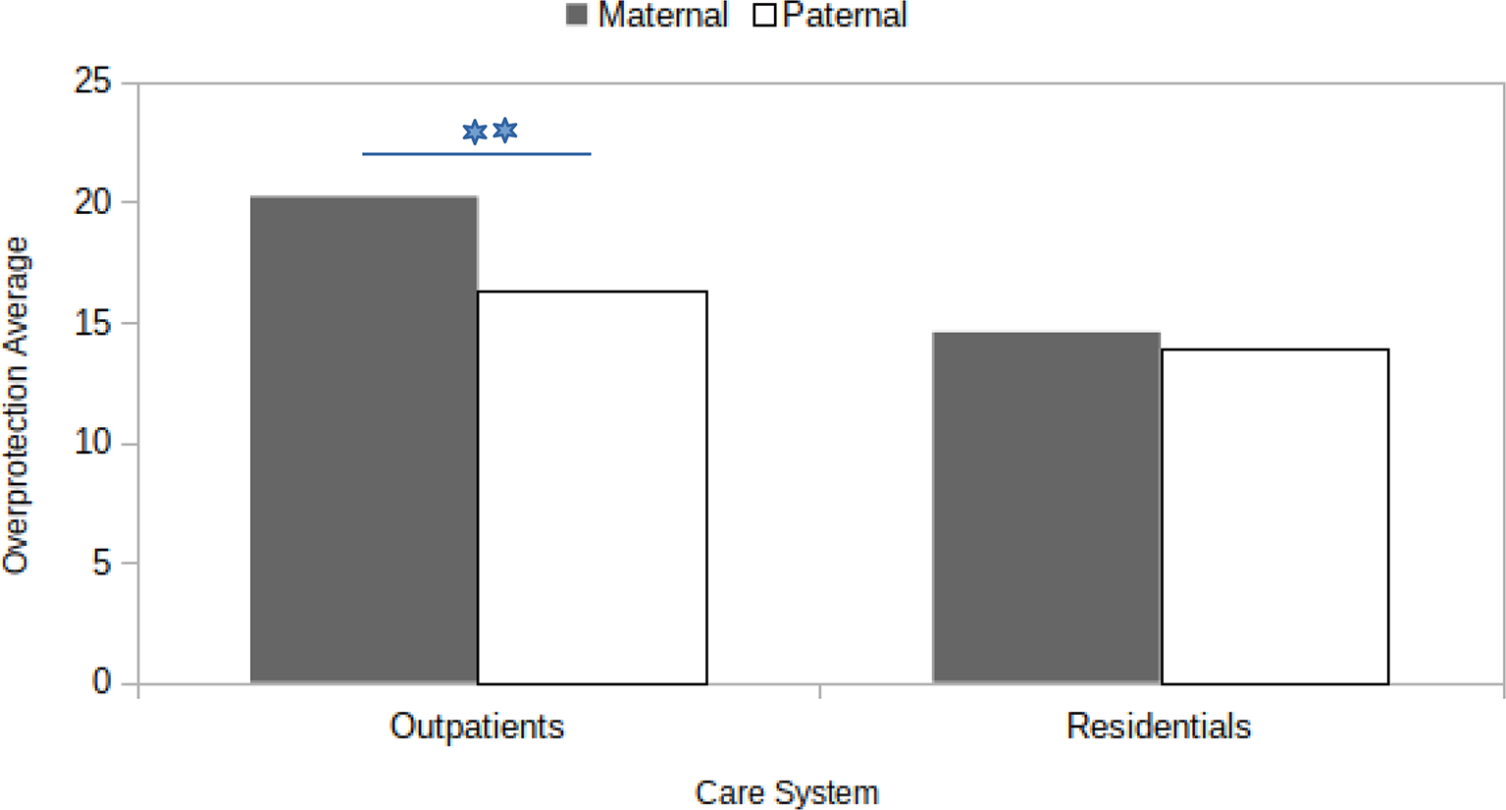
Interaction effect between (Parental Bonding Instrument) parenting style (Maternal vs Paternal) and care system (Outpatients vs Residentials) on average overprotection scores.

As the interaction effect approached statistical significance, we proceeded, exploratively, to the inspection of simple effects (**Figure 1**). Outpatient SUD reported higher levels [*F*(1, 124) = 25.373, *p* <.01, *η* ^2^ *_P_* = .17] of overprotection with respect to the mother version condition (*M* = 20.29) compared to the father version (*M* = 16.09). This difference was no more significant [*F*(1, 124) = 0.227, *p* > .05] when we considered therapeutic community SUD [overprotection: *M* (mother) = 14.60; *M* (father) = 13.90].

### Care System and Psychopathological Distress (SCL-90-R)

As the distribution of attachment categories produced qualitatively different results for the two care systems considered, we proceeded to check if the two sub-populations of subjects diagnosed with a SUD (outpatient care vs. therapeutic community care) were affected by different levels of psychopathological distress (**Table 7**). Therefore, a series of ANOVAs were performed using SCL-90-R scores on the nine psychopathology indices. The analysis of global indices is presented in the next paragraph. Not all participants completed the SCL-90-R. However, no significant relationship emerged between the chosen system of care and the missing and non-missing information of SCL-90-R [*χ* *^2^* (1) = 3.49, *p*>.05]. In general, we found no significant differences on the 9 SCL-90-R psychopathological dimensions [somatization: *F*(1,125) = 0.22, *p* = n.s.; depression: *F*(1,118) = 0.02, *p* = n.s.; anxiety: *F*(1,125) = 0.26, *p* = n.s.; hostility: *F*(1,125) = 0.37, *p* = n.s.; phobic anxiety: *F*(1,125) = 0.53, *p* = n.s.; paranoid ideation: *F*(1,125) = 0.91, *p* = n.s.; psychoticism: *F*(1,125) = 0.29, *p* = n.s.].

**Table 7:** Distribution of psychopathological risk as function of care system (Outpatients Vs Residentials) for each SCL-90-R global index: Global Severity Index (GSI), Positive Symptom Distress Index (PSDI) and Positive Symptom Total (PST).

	Outpatients				Residentials						
	Not at risk		Not at risk		Not at risk		Not at risk				
	Fr	%	Fr	%	Fr	%	Fr	%	χ^2^	df	p
GSI (N=125)^a^	67	53.6	28	22.4	21	16.8	9	7.2	.003	1	>.05
PSDI (N=93)^a^	59	63.5	6	6.5	26	27.9	2	2.1	.109	1	>.05
PST (N=120)^a^	71	59.2	21	17.5	21	17.5	7	5.8	.057	1	>.05

a not all participants completed the SCL-90-R, and the sample size for each of the three SCL-90-R indices differ as function of available information.

GSI, Global Severity Index; PSD, Positive Symptom Distress Index; PST, Positive Symptom Total; At risk, participants at risk reported a T-score > 70 at GSI, PSDI and PST; Not at risk, participants not at risk reported a T-score < 70 at GSI, PSDI and PST)

Although the two sub-populations of subjects diagnosed with a SUD did not diverge with respect to the reported psychopathological distress, we further investigated the hypothesis that there could be a difference in terms of concentration in the two care systems: people “at risk” for developing a psychopathology and those “not at risk,” considering the SCL-90-R cut-off scores indicated by 62, pp. 88–91; T scores > = 70). In this case, no significant difference emerged (**Table 7**).

### Differences in Psychopathological Distress as a Function of Care System and Attachment Categories

A series of 4 × 2 ANOVAs were performed for each of the three SCL-90-R indexes, considering the following factors: care system (outpatient care vs. therapeutic community care) and RQ attachment category system (secure vs. preoccupied vs. fearful-avoidant vs. dismissive-avoidant). A significant main effect of the RQ attachment system emerged [*F*(3, 116) = 4.83, *p* < .01, *η* ^2^ *_P_* = .11] with respect to the GSI for the preoccupied group scoring lower (*M* = .58) than both fearful-avoidant (*M* = 1.33) and dismissive-avoidant (*M* = .95) participants. The main effect of the RQ category system was also significant on the PST index [*F*(3, 112) = 3.87, *p* < .05, *η* ^2^ *_P_* = .09]; *post hoc* analysis showed lower average scores for preoccupied (*M* = 33.51) than dismissive-avoidant (*M* = 48.62) participants. None of the remaining main and interaction effects reached statistical significance.

No significant main or interaction effects were observed when the same ANOVA design was considered with PBI scores on both versions (mother vs. father) in place of the RQ attachment category system.

### Predictors of Care System Choice

Logistic regression was conducted to determine whether PBI factors (care and over-protection for both the mother and father) and RQ attachment categories (secure, preoccupied, fearful, dismissive) significantly predicted the attended care system [outpatient care (0) vs. therapeutic community care (1)]. The overall fit of the full model (constant plus all predictors at once) was statistically significant [*χ* *^2^* (7) = 37.57, *p* < .01]; this means that predictors introduced in the equation were able to reliably differentiate among outpatient SUD vs. therapeutic community SUD. The model explained about 38.9% (*Nagelkerke R* *^2^*) of the variance in group membership with a 90.5% success rate in predicting outpatient care membership and a 36.7% success rate in correctly classifying subjects diagnosed with a SUD who chose therapeutic community care. The overall success rate was 77.6%.

Interestingly, three out of five predictors (**Table 8**) reported a significant Wald coefficient; specifically, a unit increase in mother over-protection and father care significantly reduced the probability of choosing therapeutic community care [respectively *Exp(B)* = .819 and *Exp(B)* = .898]. Having a dismissive attachment style increased the probability of choosing therapeutic community care [*Exp(B)* = 4.431; meaning that dismissive people were four times more likely to choose therapeutic community care].

**Table 8:** Prediction of treatment modality, Outpatients (n = 96) and Residential (n = 30), as function of Parental Bonding Instrument’s (PBI) dimensions and Relationship Questionnaire’s attachment categories.

	Outpatients		Residential				
	M	SD	M	SD	B	Wald statistic	Exp(B)
PBI Care M	22.05	8.34	19.43	8.45	.00	.01	1.00
PBI Over-Protection M	20.19	7.03	14.60	5.88	–.20	14.11**	.82
PBI Care F	19.21	8.34	14.17	8.91	–.11	7.33**	.90
PBI Over-Protection F	16.09	7.55	13.90	9.41	.03	.80	1.03
Attachment in Close Relationship^a^	–		–			7.65 ^b^	
Preoccupied	–		–		–.22	.09	.80
Fearful/avoidant	–		–		.65	.57	1.92
Dismissing/avoidant	–		–		1.47	3.87*	4.34

^a^the reference category is that of “secure attachment”; * p<.05; ** p<.01; ^b^ p=.05

M, Mean; SD, Standard Deviation; B, logistic regression coefficient; Exp(B), odds ratio; PBI Care M, Parental Bonding Instrument Mother Care; PBI Over-Protection M, Parental Bonding Instrument Mother Over-Protection; PBI Care F, Parental Bonding Instrument Father Care; Over-Protection F, Parental Bonding Instrument Father Over-Protection; Preoccupied, Preoccupied type of Attachment in Close Relationship; Fearful/avoidant, Fearful/Avoidant type of Attachment in Close Relationship; Dismissing/avoidant, Dismissing/Avoidant type of Attachment in Close Relationship.

## Discussion

Paths to substance use and abuse are no doubt complex and involve many contextual, individual, and interpersonal variables (71). More and more studies have found a strong association between insecure attachment and emotional distress. Insecure attachment may be associated with an increase in substance use as a means of dealing with distress and negative affects (21, 30, 32, 33, 37, 38, 54, 72–74). However, data are inconsistent as to the impact of a specific quality of attachment on the development of substance dependence (75, 76).

### Current Attachment Relationships

We hypothesize that in dismissive-avoidant attachment, characterized by strong self-control and deactivation of the attachment system, substance abuse may function as a pseudo-regulator. This type of strategy—as a defensive mechanism of pretended self-sufficiency—may reduce distress and dysphoric states (21, 30, 32, 33, 54, 72, 77). In comparison, preoccupied attachment is characterized by a hyper-activation of the attachment system, thus by the need for closeness in attachment relations expressed as an exaggerated preoccupation with caregivers, together with feelings of anger and confusion. In such a case, substance abuse could reinforce family enmeshment: the family feels deeply involved by their family member’s problem. Subjects diagnosed with a SUD, therefore, would attribute a pseudo-regulatory function of his self to the family, although an extremely fragile and poorly integrated self (78, 79). The aim of this research is to contribute to the understanding of such an association, focusing on its impact on the choice of treatment modality.

Assessing attachment means of Bartholomew’s RQ, our data showed an overall higher frequency of preoccupied attachment. Considering the distribution with respect to the treatment typology, the prevailing attachment style of the outpatient subgroup was preoccupied; among therapeutic community-treated patients, the prevailing style was dismissive-avoidant. Moreover, the likelihood of choosing therapeutic community treatment increased fourfold in dismissive-avoidant subjects.

Such data are in line with the research hypothesis, according to which preoccupied subjects are more likely to be outpatients, due to their tendency to be overly involved in family relationships, from which they are not able to become autonomous. According to such a perspective, the abused substance takes the role of an external regulator (19, 80) to overcome a family’s difficulties concerning their acceptance of changes and of their son/daughter separation-individuation process.

In comparison, dismissive-avoidant subjects are more likely to be therapeutic community patients. It is plausible that this is related to their greater facility to detach from their families, keeping them at a distance. On the other hand, dismissive-avoidant subjects may be in need of a support that may replace the substance as an external emotional regulator to compensate for their lack of modulation and response to their internal needs (21, 30, 32, 33, 54).

The hypothesis of a higher frequency of the fearful-avoidant category, as shown in the literature (38, 54), was not confirmed. Fearful individuals cannot deactivate their attachment system under distress; in such conditions, they perceive anxiety as linked to attachment, as preoccupied subjects do, but at the same time they are unable to look for and eventually obtain closeness to the significant figure. In our sample, instead, dismissive-avoidant subjects prevailed; through deactivation, they seemed to have an organized strategy to deal with stress (81). However, the literature highlights that fearful attachment plays a substantial role especially in the chronicization of abuse; our study has no adequate data on the issue (36, 82). In addition, our data concerned adult patients, whereas many findings refer to adolescents and college students. Certainly, an individual’s developmental stage may influence his or her perception of attachment experiences and relations.

Finally, it is worth mentioning that a portion of the sample showed secure attachment patterns. It is important to acknowledge that self-reported evaluations may be insufficient to understand the role of attachment in individuals with a diagnosis of SUD. Undoubtedly, attachment is conceived as a largely unconscious process that could be better explained by means of implicit measures.

#### Past Attachment Relationships

For a better understanding of such outcomes, we also evaluated attachment through the PBI. The PBI allowed us to evaluate the quality of attachment with respect to each parent. Specifically, there was an over-representation of the category “affectionless control” with respect to mothers and an under-representation of the category “affectionate constraint” with respect to fathers. Certainly, inadequate parenting has been associated with difficulties in coping with stress and with more frequent negative feelings and behaviors (83, 84). Moreover, the results revealed that care and overprotection had higher mean scores in the outpatient treated subgroup compared to the therapeutic community patients. In particular, there was a significantly higher score regarding maternal overprotection among patients attending outpatient care, whereas a weak or absent bond as regards mothers emerged among individuals who attended a therapeutic community. From such findings, the attachment experiences with mothers seem to play a crucial role. Indeed, the perceived parental bonding and the representations of attachment are linked to the emotional development of the individual and to her/his ability to regulate inner affects and emotions (29, 85). Several researchers have shown that infants develop emotion regulation in the context of early mother-infant interactions (86–88) Maternal unavailability or unpredictability contribute to dysregulation because the mother does not support adequate stimulation nor arousal regulation for her child (89). Lyons-Ruth suggests that the context of the attachment relationship provides the fundamental roots of these processes, that is intersubjectivity “an essential function of mind” (90).

#### Self-Reported Symptoms

It is also important to consider the complexity of the psychological, mental organization of our sample. Indeed, substance abuse is characterized by high levels of comorbidity, which may affect 90.0% of subjects diagnosed with a SUD (91). The most frequent associations are with mood disorders, anxiety disorders, and personality disorders (92–95). Our study does not consider this variable. However, no difference emerged with respect to self-perceived symptomatology (SCL-90-R, 96) between the two subgroups. Indeed, psychopathology has no direct reference to the attachment motivational system (4, 97, 98); rather, the expression of symptoms is the outcome of a complex, multifactorial process, in which innate predispositions, learned behaviors, and context specificities all play an important role. Nevertheless, regardless of the treatment modality, both fearful-avoidant and dismissive-avoidant individuals, who are characterized by a more disruptive and disorganized representation of themselves, reported higher self-perceived symptomatology, in line with previous research (81, 99).

## Limitations

Despite their significance, our results call for caution. The relatively small size of the sample, the effect sizes ranging from low to medium values, and the lack of a control group limit the generalizability of our findings and need to be replicated in order to verify the significant effects that we found.

As well, the use of self-reports—which rely on a subject’s personal views of himself or herself and of his or her caregivers—does not allow to give a complete good definition of our clinical sample. Besides, the diagnoses were provided by the Local Health Service, with no further check on behalf of the research team.

Moreover, as mentioned above, comorbidity should be considered in future studies. Undeniably, the comorbidity of personality disorders and other severe disturbances may affect the course and prognosis of a SUD as well as its treatment outcome. In the same direction, the specific effect of the types of used substances as well as the differential impact of abuse and dependence should also be included in future studies.

Finally, the current study has not included the assessment of multiple attachments, which may play an important protective factor in the context of personality development (100). Information on the growing family type should also be included in future studies to fully understand the complex role of attachment relationships in the development of such disorders.

## Conclusion

This study further confirmed the importance of attachment quality when planning interventions programs to support significant relationships (52, 55, 101–104).

For a deeper comprehension of the dynamics of attachment within individuals diagnosed with SUD, additional longitudinal studies are required to assess mental representations of attachment experiences at the beginning and end of the intervention. Such studies will provide more clear data concerning the stability and changes of internal working models of attachment after treatment.

It is important not to consider substance abuse as equivalent to an attachment disorder, as this is simplistic and reductive. The different distribution of attachment styles in relation to the typology of a care system may promote therapeutic compliance and consequently more adequate and efficacious interventions, corresponding to the individual and the context of his or her life and development.

## Data Availability Statement

The datasets generated for this study are available on request to the corresponding author.

## Ethics Statement

The Ethical Committee of the University of Cagliari approved this research with the protocol n. 2019-UNCACLE-0228682. Written informed consent was obtained from all participants.

## Author Contributions

LV helped prepare the study design, coded the instruments, and wrote all sections of the manuscript. FP prepared the data set, performed statistical analyses, prepared tables and figures, and contributed to the method and results sections. MA helped prepare the study design, organized the recruitment of the sample, and supervised data collection and the research team. MB and RR contributed to the recruitment of the sample and data collection. All authors reviewed and approved the manuscript for publication.

## Funding

This work was supported by the Open Access Publishing Fund of the University of Cagliari, with the funding of the Regione Autonoma della Sardegna - L.R. n. 7/2007.

## Conflict of Interest

The authors declare that the research was conducted in the absence of any commercial or financial relationships that could be construed as a potential conflict of interest.
